# Hypertonic saline reduces lipopolysaccharide-induced mouse brain edema through inhibiting aquaporin 4 expression

**DOI:** 10.1186/cc11670

**Published:** 2012-10-04

**Authors:** Cao C, Yu X, Liao Z, Zhu N, Huo H, Wang M, Ji G, She H, Luo Z, Yue S

**Affiliations:** 1 Department of Pediatrics, Xiangya Hospital, Central South University, Xiangya Road, Changsha, Hunan, China 410008; 2 Department of Physiology, Xiangya Medical School, Central South University, Xiangya Road, Changsha, Hunan, China 410013; 3 Department of Pharmacology, Emory University, 1518 Clifton Road Northeast, Atlanta, GA, USA GA 30322

**Keywords:** hypertonic saline, lipopolysaccharide, brain edema, aquaporin 4, protein kinase C, interleukin-1 beta

## Abstract

**Introduction:**

Three percent sodium chloride (NaCl) treatment has been shown to reduce brain edema and inhibited brain aquaporin 4 (AQP4) expression in bacterial meningitis induced by *Escherichia coli*. Lipopolysaccharide (LPS) is the main pathogenic component of *E. coli*. We aimed to explore the effect of 3% NaCl in mouse brain edema induced by LPS, as well as to elucidate the potential mechanisms of action.

**Methods:**

Three percent NaCl was used to treat cerebral edema induced by LPS in mice *in vivo*. Brain water content, IL-1β, TNFα, immunoglobulin G (IgG), AQP4 mRNA and protein were measured in brain tissues. IL-1β, 3% NaCl and calphostin C (a specific inhibitor of protein kinase C) were used to treat the primary astrocytes *in vitro*. AQP4 mRNA and protein were measured in astrocytes. Differences in various groups were determined by one-way analysis of variance.

**Results:**

Three percent NaCl attenuated the increase of brain water content, IL-1β, TNFα, IgG, AQP4 mRNA and protein in brain tissues induced by LPS. Three percent NaCl inhibited the increase of AQP4 mRNA and protein in astrocytes induced by IL-1β *in vitro*. Calphostin C blocked the decrease of AQP4 mRNA and protein in astrocytes induced by 3% NaCl *in vitro*.

**Conclusions:**

Osmotherapy with 3% NaCl ameliorated LPS-induced cerebral edema *in vivo*. In addition to its osmotic force, 3% NaCl exerted anti-edema effects possibly through down-regulating the expression of proinflammatory cytokines (IL-1β and TNFα) and inhibiting the expression of AQP4 induced by proinflammatory cytokines. Three percent NaCl attenuated the expression of AQP4 through activation of protein kinase C in astrocytes.

## Introduction

Hypertonic saline (HS) has been widely used in the treatment of patients with brain edema resulting from cerebral infarction, hemorrhage or traumatic brain injury [1-8], and the therapeutic efficiency of HS has also been proved by animal studies [9-13]. Previous reports suggested that HS was more effective in treating brain edema resulting from cerebral hemorrhage, ischemic or traumatic brain injury, as compared with equiosmolar doses of mannitol, in animal studies [9,12] and clinical trials [14-17]. Our previous study confirmed that adjunctive 3% (1,026 mOsm/L) NaCl treatment reduced brain edema and attenuated brain damage with a superior effect over 20% mannitol in a rabbit bacterial meningitis model [11]. These studies indicated that HS has superior action to mannitol in the treatment of brain edema, in addition to its osmotic effect.

However, little was known of the action of HS besides its osmotic effect in the treatment of brain edema. HS has been suggested to be superior to mannitol as a hyperosmolar agent because it augments intravascular volume and cardiovascular performance in addition to causing 'dehydration' of the brain. In both animal and human studies, HS has been shown to produce a prolonged increase in intravascular volume and plasma volume expansion. In doing so, mean arterial pressure is increased and cerebra1 perfusion pressure is improved [9,18]. HS has also been shown to have anti-inflammatory effects, which, in turn, modulate blood-brain barrier (BBB) permeability [7,19]. Zeynalov *et al. *reported that HS attenuates BBB disruption depending on the presence of perivascular aquaporin 4 (AQP4) in post-ischemic cerebral edema[20]. AQP4 is the primary water channel found in the brain, which is expressed in astrocyte foot processes, in ependymal cells, and in subependymal astrocytes [21,22]. The expression of AQP4 is up-regulated in wild type mice and AQP4 null mice have significantly less brain edema in water intoxication cerebral edema, ischemic stroke and pneumococcal meningitis [23,24]. This suggests that AQP4 plays an important role in brain edema formation. Zeng *et al. *reported that 10% NaCl can down-regulate expression of AQP4 in perivascular astrocytes in a rat cerebral ischemic edema model [25]. Recently, we reported that 3% NaCl inhibited the up-regulation of brain AQP4 protein expression in bacterial meningitis induced by *Escherichia coli *in rabbits [11]. However, the mechanism of HS down-regulation of AQP4 expression still remains unclear. Lipopolysaccharide (LPS) is the main pathogenic component of *E.coli *Gram-negative bacterium. Administration of LPS to animals causes pathogenesis, mimicking what occurs in patients [26,27]. LPS can induce cerebral edema formation and up-regulate expression of brain AQP4 in the mouse [28]. Investigating the effect of HS on up-regulation of brain AQP4 during LPS-induced mice brain edema would provide clues to reveal the mechanism of HS down-regulation of brain AQP4 in bacterial meningitis.

Protein kinase C (PKC) is a family of serine- and threonine-specific protein kinases and plays an important role in the regulation of AQP4 expression. Activation of PKC with phorbol 12, 13-dibutyrate reduces the AQP4 water permeability of LLC-PK1 cells transfected with AQP4 cDNA constructs [29]. Treatment of rat astrocytes with phorbol ester 12-O-tetradecanoylphorbol 13-acetate, a PKC activator, causes a decrease in AQP4 mRNA and protein, which can be inhibited by PKC inhibitors [30]. AQP4 up-regulation and brain edema formation were attenuated by phorbol 12-myristate 13-acetate, a PKC activator, in a rat cerebral ischemia model [31-33]. HS increases total PKC activity and induces PKCα, PKCd, and PKCe translocation from the cytosol to the membrane in NIH/3T3 cells [34]. We hypothesized that activation of PKC would contribute to down-regulation of AQP4 expression induced by HS.

Thus, in the present study we investigated the effects of 3% NaCl on brain AQP4 expression in cerebral edema induced by LPS. Additionally, we also investigated the role of PKC in mediating the down-regulation of AQP4 expression induced by HS in primary astrocytes.

## Materials and methods

### Animals and experimental groups

**Figure 1:**
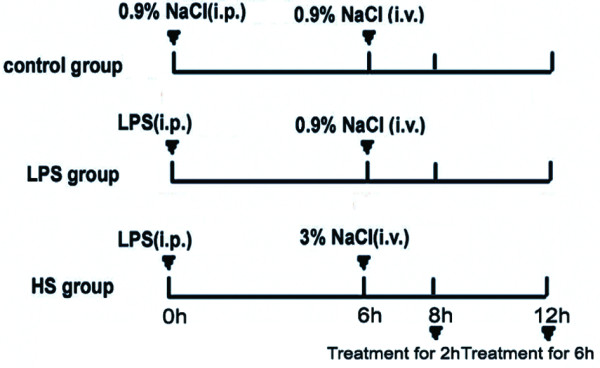
**Experiment design**. All animals were randomly divided into control group, lipopolysaccharide (LPS) group and hypertonic saline (HS) group. The LPS group was given LPS as a single injection of 10 mg/kg i.p.. The HS group was given 3% NaCl as a single injection of 20 ml/kg i.v. through the tail vein at 6 hours following injection of LPS, and the control group received 0.9% NaCl as control. The mice were sacrificed at 8 hours or 12 hours after injection of LPS and their brains harvested.

KunMing mice were used for experiments at four weeks of age. The animals were maintained with 12-hour light and dark cycles with free access to food and water. The experimental design is shown in Figure 1. Mice were randomized into a control group, a LPS group and a HS group. The LPS group was given LPS (Sigma, Santa Clara, CA USA) as a single injection of 10 mg/kg i.p.. The HS group was given 3% NaCl as a single injection of 20 ml/kg i.v. through the tail vein at 6 hours following injection of LPS, and the control group received 0.9% NaCl as control. The mice were sacrificed at 8 hours or 12 hours after injection of LPS. The animals were maintained and experiments were performed in accordance with the guidelines set by the Institutional Animal Care and Use Committee of University of Central South University. This research protocol was approved by the Ethics Committee of Xiangya Hospital of Central South University.

### Isolation and treatment of astrocytes

Astrocytes were isolated from newborn Sprague-Dawley rats, using a method described by Nicholson and Renton [35]. Briefly, after removal of the meninges, brains of newborn Sprague-Dawley rats were disaggregated using nylon sieves (120 mm pore size) and seeded in 50-ml tissue culture flasks in (D)MEM containing 20% heat-inactivated FCS. Growth medium was replaced on the third and sixth day with (D)MEM containing 20% FCS. On day 10, subcultures were made by treating the cells with 0.25% trypsin:0.25% ethylenediaminetetraacetic acid (EDTA) and, after washing, 1 × 10^6 ^cells/ml plated in a 50-ml tissue culture flask in (D)MEM with 10% FCS. After a two-week incubation, cultures were approximately 90% to 95% confluent and contained predominantly astrocytes, with a minor contribution of microglia and oligodendrocytes (3% and 2%, respectively) as determined by immunocytochemistry using antibodies directed against the astrocyte marker glial fibrillary acid protein [36]. Then, the cells were randomly divided into control group, IL-1β group, IL-1β + HS group, IL-1β + calphostinC group and IL-1β + calphostinC + HS group. IL-1β group cells were incubated for six hours with fresh serum-free (D)MEM, containing IL-1β (5 ng/mL) (Recombinant Rat Interleukin-1 beta, ProSpec-Tany TechnoGene Ltd, Rehovot, Israel). IL-1β + HS group cells were incubated with fresh serum-free (D)MEM (containing IL-1β 5 ng/mL) for 3 hours, 3% NaCl for 15 minutes and fresh serum-free (D)MEM (containing IL-1β 5 ng/mL) for 3 hours. Treatment of IL-1β + calphostin C + HS group cells was the same as IL-1β + HS group cells, except for pretreatment with calphostin C (5 uM; Sigma) for 30 minutes before 3% NaCl. The control group received 0.9% NaCl instead of 3% NaCl and the fresh serum-free (D)MEM.

### Brain water content

The brains were removed from the mice at the end of the experiment, weighed immediately and then kept at 106°C for 72 hours. The brains were weighed during this process at 48 hours and then again at 72 hours to make sure they had reached a consistent weight. Brain edema was estimated by comparing wet to dry weight ratios. Tissues were weighed with a scale to within 0.001 mg. The percentage of brain water in the tissue was calculated as (wet weight - dry weight) ×100/wet weight.

### Detection of IL-1β, TNFα protein and IgG by ELISA

Briefly, animals were sacrificed at the end of the experiment and the whole brain from each animal was collected and weighed immediately. The brain tissue was homogenized with a glass homogenizer with the ratio of 100 mg tissue:1 ml ice-cold PBS (pH 7.4) and centrifuged at 12,000 g for 20 minutes at 4°C. IL-1β, TNFα protein and IgG in the brain tissues were detected by ELISA, following the manufacturer's instructions(**Mouse **IL-1β ELISA Kit/**Mouse **TNFα ELISA Kit/**Mouse **IgG ELISA Kit)(R&D Systems Inc., Minneapolis, MN USA). Data were acquired using a 96-well plate reader. The contents are expressed as pg cytokines/g tissue or ng IgG/g tissue.

Brain edema could be due to both vasogenic and cytotoxic changes. The destruction of the BBB leads to vasogenic cerebral edema. To gain insight into this possibility, the BBB integrity was assessed by analyzing plasma IgG extravasation via ELISA. IgG is a plasma protein which exists mainly in the vascular. Abnormal permeability to IgG occurs after breakdown of the BBB and an increase of IgG in brain tissues can indicate breakdown of the BBB [37,38].

### Western blotting

Proteins were extracted from brain tissue using a total protein extraction kit (Pulilai Gene Technology Co., Ltd., Beijing, China) according to the manufacturer's protocol. Samples of supernatants containing protein were heated to 100°C for 5 minutes. Protein bands were electroblotted onto polyvinylindene difluoride membranes (Millipore, Boston, Massachusetts, USA). After transfer, the membranes were blocked with 5% nonfat milk in tris-buffered saline for 0.5 hours, and then incubated with the primary antibodies according to the manufacturer's recommendations. The primary antibodies used were as follows: AQP4 (rabbit polyclonal antibody 1:200, Beijing Biosynthesis Biotechnology Co., Ltd, Beijing, China), GAPDH (rabbit polyclonal antibody 1:3,000, Nanjing Genscript Corporation, Nanjing, China). After being washed three times with tris-buffered saline with 0.1% Tween-20, the membranes were incubated with the horseradish peroxidase (HRP)-conjugated secondary antibodies (1:5,000)(Nanjing Genscript Corporation) for 1 hour. Bands were visualized using the ECL kit per the manufacturer's manual(LumiGOLD ECL Western Blotting Detection Kit, SignaGen Laboratories, Gaithersburg, MD USA). The signal intensity of AQP4 levels in each group (*n *= 4) was measured.

### Real-time RT-PCR

Total RNA was extracted from brain tissues or cells with trizol reagent (ShineGene, Shanghai, China) and contaminating genomic DNA was removed with RNasefree DNase I (ShineGene). RT-PCR was performed using a first-strand cDNA synthesis kit (ShineGene) following the manufacturer's instructions.

Quantitative RT-PCR was carried out on an FTC2000 real-time PCR system using a FastStart DNA Master plus SYBR Green I kit (ShineGene) following the manufacturer's instructions. The primers used were: AQP4(213 bp), Forward: GCTTTCTGGAAGGCAGTCTCA, Reverse: GGCTACAGTCACAGCGGGA; IL-1(196 bp), Forward: GCTTCAGGCAGGCAGTATCA, Reverse: TGCAGTTGTCTAATGGG AACG; TNFα(156 bp), Forward: TACTGAA CTTCGGGGTGATCG, Reverse: CCACTTGGTGGTTTGCTACG; β-actin(263 bp), Forward: GAGACCTTCAACACCCCAGC, Reverse: ATGTCACGCACGATTTC CC(Mouse); AQP4(138 bp), Forward: CGC CAAGTCCGTCTTCTACA, Reverse: GCCAGCAGTGAGGTTTCCAT, β-actin(150 bp), Forward: CCCATCTATGAGGGTTACGC, Reverse: TTTAATGTCACGCACGA TTTC(rat). The first segment of the amplification cycle consisted of a denaturation program at 94°C for 4 minutes. The second segment consisted of denaturation, primer annealing, elongation and a quantification program repeated for 35 cycles. The third segment consisted of a melting curve program. The final segment consisted of a cooling program at 72°C. The expression of target genes was measured in tetramerous, and was normalized to β-actin. Gene expression was quantified using a modification of the 2(-Delta Delta C(T)) method as previously described[39].

### Flow cytometry

With direct staining, astrocytes were incubated with AQP4-PE (Santa Cruz Biotechnology, Santa Cruz, CA USA) antibody, a monoclonal antibody conjugated to a fluorochrome phycoerythrin. This procedure was quick and direct; it merely involved a half-hour incubation of cells with antibody (at 4°C), followed by several washes to remove weakly or nonspecifically bound antibodies. Cells thus treated were ready for flow analysis (although final fixation with 1% electron microscopic-grade formaldehyde provided a measure of biological safety and long-term stability). The mean fluorescence intensity of cells was measured in tetramerous with a flow cytometer (Beckman Coulter Co., Caguas, PR USA).

### Statistical analysis

All values were presented as mean ± S.E.M. Differences in various groups were determined by one-way analysis of variance. A value of *P *< 0.05 was considered statistically significant.

## Results

### Three percent NaCl attenuated brain edema induced by LPS

**Figure 2:**
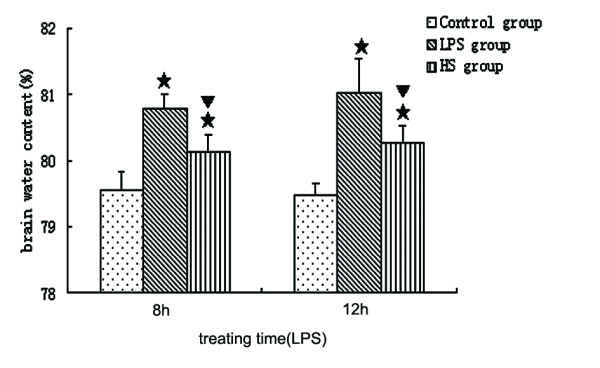
**Brain water content in LPS group, HS group, and control group**. The percentage of brain water content was significantly increased in LPS-treated mice at 8 hours and 12 hours following LPS injection when compared with the corresponding controls. However, treatment with 3% NaCl attenuated the increase in brain water content at 8 hours and 12 hours after LPS injection. (single star) *P *< 0.05 versus control group. (single triangle) *P *< 0.05 versus LPS group. HS, hypertonic saline; LPS, lipopolysaccharide.

**Figure 3:**
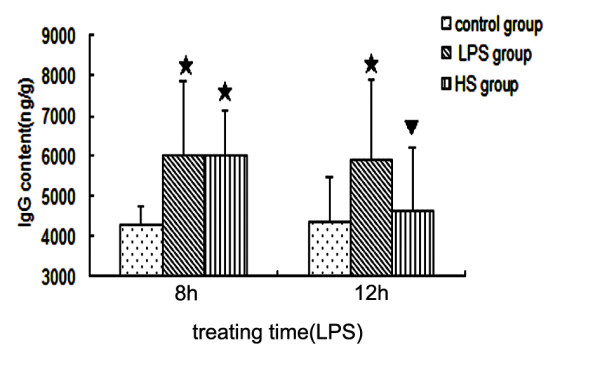
**Detection of IgG using ELISA in the brain tissues in the LPS group, HS group, and control group**. IgG increased in LPS-treated mice at 8 hours and 12 hours following LPS injection, compared with corresponding controls. However, after treatment with 3% NaCl, IgG significantly decreased at 12 hours after LPS injection. (single star) *P *< 0.05 versus control group. (single triangle) *P *< 0.05 versus LPS group. ELISA, enzyme-linked immunosorbent assay; HS, hypertonic saline; IgG, immunoglobulin G; LPS, lipopolysaccharide.

To determine whether edema occurred in the brains of mice administered LPS, we measured the water content in the brains of control and LPS-treated mice. The brain water content (BWC) was significantly increased in LPS-treated mice at 8 hours and 12 hours following LPS injection when compared with corresponding controls (Figure 2). Treatment with 3% NaCl attenuated the increase in BWC at 8 hours and 12 hours after LPS injection (Figure 2). As shown in Figure 3, IgG in brain tissues increased in LPS-treated mice at 8 hours and 12 hours following LPS injection, but after treatment with 3% NaCl IgG significantly decreased at 12 hours after LPS injection, compared with LPS-treated mice. The results suggest that 3% NaCl alleviated the breakdown of the BBB induced by LPS.

### Three percent NaCl inhibited the increase of IL-1β and TNFα in brain tissues induced by LPS

**Figure 4:**
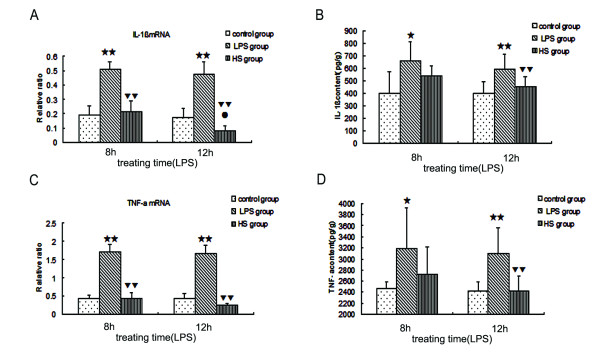
**Expression of IL-1β and TNFα in the brain tissues in LPS group, HS group, and control group**. IL-1β (**a**) and TNFα (**c**) mRNA was measured using real-time RT-PCR. β-actin was also included in the PCR reaction as a reference. IL-1β (**b**) and TNFα (**d**) proteins were measured using ELISA. (a, c) IL-1β and TNFα mRNA increased in LPS-treated mice at 8 hours and 12 hours following LPS injection, compared with the corresponding controls, but they significantly decreased in the mice at 8 hours and 12 hours following LPS injection after treatment with 3% NaCl. (b, d) IL-1β and TNFα protein increased in LPS-treated mice at 8 hours and 12 hours following LPS injection, compared with the corresponding controls, but it significantly decreased in the mice at 12 hours following LPS injection after treatment with 3% NaCl for 6 hours. (single star) *P *< 0.05, (two stars) *P *< 0.01 versus control group. (single triangle) *P *< 0.05, (two triangles) *P *< 0.01 versus LPS group. (single circle) *P *< 0.05, versus 8 hour group. ELISA, enzyme-linked immunosorbent assay; HS, hypertonic saline; IL-1β, interleukin-1beta; LPS, lipopolysaccharide; RT-PCR, reverse transcriptase-polymerase chain reaction; TNFα, tumor necrosis factor alpha.

IL-1β and TNFα are two important proinflammatory cytokines. As shown in Figure 4 (a, c), IL-1β and TNFα mRNA in brain tissues increased at 8 hours and 12 hours following LPS injection, compared with corresponding controls, but it significantly decreased in the mice at 8 hours and 12 hours following LPS injection after treatment with 3% NaCl. The ELISA results showed that the content of both IL-1β and TNFα in brain tissues was significantly increased at 8 hours and 12 hours following LPS injection, but IL-1β and TNFα significantly decreased in the mice at 12 hours following LPS injection after treatment with 3% NaCl for 6 hours (Figure 4b, d). The results indicate that treatment with 3% NaCl for 2 hours reduced the increase of IL-1β and TNFα mRNA in the brain tissues induced by LPS, and treatment with 3% NaCl for 6 hours further reduced the increase of IL-1β and TNFα protein in the brain tissues induced by LPS.

### Three percent NaCl blocked the increase of AQP4 mRNA and protein in brain tissues induced by LPS *in vivo *

**Figure 5:**
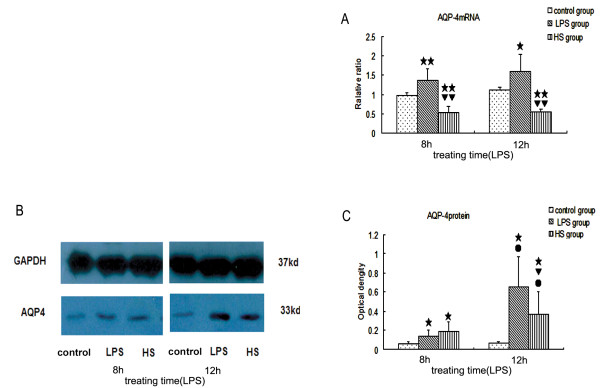
**AQP4 mRNA and protein expression in the brain tissues of the LPS group, HS group, and control group**. (**a**) AQP4 mRNA increased in LPS-treated mice at 8 hours and 12 hours following LPS injection, compared with the corresponding controls. However, it significantly decreased in the mice at 8 hours and 12 hours following LPS injection after treatment with 3% NaCl. β-actin was also included in the PCR reaction as a reference. (**b**) Shows AQP4 (33kD) and GAPDH (37kD) immunoreactive bands, respectively. (c)The optical density of AQP4 increased in LPS-treated mice at 8 hours and 12 hours following LPS injection, when compared with the corresponding controls. However, the optical density of AQP4 significantly decreased in the mice at 12 hours following LPS injection after treatment with 3% NaCl for 6 hours. (single star) *P *< 0.05, (two stars) *P *< 0.01 versus control group. (single triangle) *P *< 0.05, (two triangles) P < 0.01 versus LPS group. (single circle) *P *< 0.05, versus 8 hour group. AQP4, aquaporin 4; HS, hypertonic saline; LPS, lipopolysaccharide; PCR, polymerase chain reaction.

AQP4 expression was determined at the mRNA level by real-time RT-PCR and at the protein level by Western blotting. AQP4 mRNA and protein in brain tissues were significantly increased in LPS-treated mice at 8 hours and 12 hours following LPS injection, compared with the corresponding controls (Figure 5). After treatment with 3% NaCl, AQP4 mRNA was significantly decreased in brain tissues at 8 hours and 12 hours following LPS injection, compared with LPS-treated mice. AQP4 protein in the HS group was lower than in the corresponding LPS group at 12 hours following LPS injection after treatment with 3% NaCl for 6 hours (Figure 5). The results indicate that LPS can up-regulate AQP4 mRNA and protein expression in brain tissues, and 3% NaCl can inhibit AQP4 expression and reduce AQP4 mRNA and protein levels in brain tissues induced by LPS.

### Three percent NaCl blocked the increase of AQP4 mRNA and protein in the primary astrocytes induced by IL-1β through activation of PKC *in vitro *

**Figure 6:**
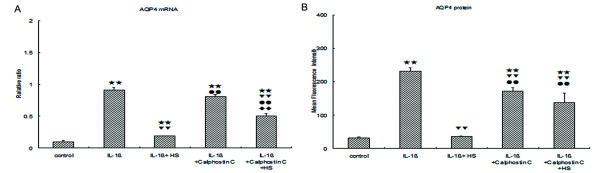
**AQP4 mRNA and protein expression in the astrocytes *in vitro***. (**a**) AQP4 mRNA increased in IL-1β-treated astrocytes, when compared with the corresponding controls and AQP4 mRNA significantly decreased in the IL-1β + 3% NaCl-treated astrocytes. However, AQP4 mRNA significantly increased in the IL-1β + calphostinC + 3% NaCl-treated astrocytes, compared with IL-1β + 3% NaCl-treated astrocytes. β-actin was also included in the PCR reaction as a reference. (**b**) The mean fluorescence intensity of AQP4 increased in IL-1β-treated astrocytes, compared with corresponding controls and the mean fluorescence intensity of AQP4 significantly decreased in the IL-1β + 3% NaCl-treated astrocytes. However, the mean fluorescence intensity of AQP4 significantly increased in the IL-1β + calphostin C + 3% NaCl-treated astrocytes, when compared with IL-1β + 3% NaCl-treated astrocytes. (two stars) *P *< 0.01 versus control. (two triangles) *P *< 0.01 versus IL-1β. (two circles) *P *< 0.01 versus IL-1β + HS. (two diamonds) *P *< 0.01 versus IL-1β + calphostin C. AQP4, aquaporin 4; HS, hypertonic saline; IL-1β, interleukin-1beta; PCR, polymerase chain reaction.

IL-1β is one of the major proinflammatory cytokines induced by LPS, and it can cause up-regulation of AQP4 in primary astrocytes [40]. To determine whether 3% NaCl could directly antagonize the IL-1β action, we investigated the effect of 3% NaCl on up-regulation of AQP4 induced by IL-1β. As shown in Figure 5, IL-1β significantly increased AQP4 mRNA expression as determined by real-time-PCR and membrane protein level as determined by flow cytometry in IL-1β-treated astrocytes. Three percent NaCl significantly decreased AQP4 mRNA and membrane protein levels in the IL-1β + 3% NaCl -treated astrocytes (Figure 6). In order to observe the possible mechanism of 3% NaCl down-regulation of the expression of AQP4, we investigated the effect of calphostin C, a specific inhibitor of PKC, on the down-regulation of AQP4 induced by 3% NaCl. Pretreatment with calphostin C attenuated the decrease of AQP4 mRNA and protein in the primary astrocytes induced by 3% NaCl (Figure 6).

## Discussion

LPS is the critical pathogenic component of gram-negative bacteria. Administration of LPS to rats or mice induces up-regulated expression of IL-1β and TNFα in brain tissues, disruption of the BBB and brain edema formation [28,41-43]. Brain edema has traditionally been classified into three major types: cytotoxic, vasogenic and interstitial [44]. The mechanisms of brain edema induce by LPS are not fully elucidated. The proinflammatory cytokines of IL-1β and TNFα and complement cascade may play an important role in development of brain edema induced by LPS [28,41,43]. Hypertonic solutions are a mainstay of osmotherapy for cerebral edema. The accepted view is that the potent anti-edema effect of HS is affected primarily via egress of water from the interstitial and extracellular space into the intravascular compartment through an intact BBB [11,45,46]. In addition to this primary action, HS has been shown to exert beneficial non-osmotic cerebral effects. Our previous study confirmed that adjunctive 3% NaCl treatment inhibited the expression of inflammatory factors in a rabbit bacterial meningitis model [11]. Our results confirm that intraperitoneal injection of LPS induced up-regulated expression of IL-1β and TNFα, disruption of the BBB and brain edema. Adjunctive 3% NaCl treatment for 2 hours could effectively reduce brain edema induced by LPS. After treatment with 3% NaCl for 6 hours, IL-1β and TNFα mRNA and protein, and IgG in brain tissues decreased significantly and brain water content did not appear to rebound. We further confirmed that in addition to its osmotic force, 3% NaCl could inhibit expression of IL-1β and TNFa, alleviate disruption of the BBB and reduce cerebral edema induced by LPS.

AQP4 is a water-channel protein predominantly expressed in astrocyte foot processes at the borders between the brain parenchyma and major fluid compartments, and in ependymal cells lining the ventricles in contact with cerebrospinal fluid in the brain [22,47]. This distribution suggests that AQP4 probably participates in water fluxes into and out of the brain parenchyma. AQP4 is up-regulated in the brain in ischemic brain edema [48,49]. AQP4 probably plays a critical role in reabsorption of cerebrospinal fluid and regulation of brain edema [50]. AQP4 null mice have lower mortality and are less prone to cytotoxic brain edema, including water intoxication cerebral edema [23], early focal cerebral ischemia [23] and bacterial meningitis [24]. Accordingly, overexpression of AQP4 in transgenic mice accelerates progression of cytotoxic brain swelling [51]. Down-regulation of AQP4 could be a way in which HS ameliorates cerebral edema [11,20,25]. AQP4 down-regulation slows the rate of water entry into the brain in cytotoxic edema. Our results show that injection of LPS could up-regulate expression of AQP4 mRNA and protein, which is consistent with previous reports [28,37,43]. Our results also showed for the first time that 3% NaCl could down-regulate expression of AQP4 mRNA and protein in the brain edema induced by LPS. This may be another non-osmotic mechanism of HS in the treatment of cerebral edema induced by LPS.

The mechanism of HS down-regulation of AQP4 expression in cerebral edema is not yet known. Different authors have observed that HS could down-regulate the expression of AQP4 in cerebral edema induced by stroke [12], bacterial meningitis [11] or local cryoinjury [52]. However, they did not study the mechanism of HS down-regulation of AQP4 expression in cerebral edema. Proinflammatory cytokines are released as an important part of LPS induced brain injury. Previous study has shown that IL-1β induces the expression of AQP4 in primary astrocytes [40]. It is also reported that TNFα is a key mediator of brain edema formation, disruption of the BBB and up-regulation of AQP4 expression induced by LPS [28]. These studies suggest that LPS induced up-regulated expression of AQP4 probably through increasing the production of IL-1β or TNFα. Cuschieri *et al. *reported that HS preconditioning reduces TNFα production induced by LPS in alveolar macrophages [53]. Present results show that 3% NaCl treatment reduced the up-regulation of AQP4 expression and reduced the production of IL-1β and TNFα induced by LPS *in vivo*. All of the above suggest that 3% NaCl induced down-regulation of AQP4 expression and attenuated disruption of the BBB induced by LPS, probably through limiting the production of proinflammatory cytokines (IL-1β and TNFα) in LPS-induced brain edema.

Astrocytes are the main cell type that swell in cytotoxic brain edema [54] and the cells are also the main cell type that express AQP4 in the brain [21]. We further investigated the effect of HS on AQP4 expression induced by IL-1β in astrocytes *in vitro *to explore the mechanism of HS down-regulation of brain AQP4. The results show that IL-1β significantly increased the AQP4 mRNA and membrane protein level in the astrocytes *in vitro *and that 3% NaCl attenuated the increase of AQP4 mRNA and protein induced by IL-1β. These results indicate that 3% NaCl also directly antagonizes the IL-1β action of inhibiting up-regulated expression of AQP4 in the astrocytes. This may be another mechanism by which 3% NaCl inhibits the up-regulation of AQP4 expression induced by LPS.

PKC serves as a second messenger for G-protein receptors that are coupled to the phosphoinositide pathway, causing either a transient rise in intracellular Ca^2+^, through inositol-triphosphate or activating PKC through diacylglycerol. Previous studies have shown that activation of PKC decreases the expression of AQP4 in rat astrocytes [30] and attenuates AQP4 up-regulation and brain edema formation [31-33].

Pretreatment of the cultured rat astrocytes with cycloheximide (an inhibitor of protein synthesis) or actinomycin D (an inhibitor of transcription) does not change the decrease in AQP4 mRNA induced by the phorbol ester 12-O-tetradecanoylphorbol 13-acetate [55]. It is suggested that PKC may be involved in AQP4 regulation on the transcriptional level, rather than affecting *de novo *protein synthesis or the stability of AQP4 mRNA. Tang *et al. *reported that thrombin inhibits AQP4 expression through a PKC-dependent pathway in cultured astrocytes [56]. It is reported that 3% NaCl can activate PKC [34]. PKC mu performs a critical function in hypertonicity-induced heat shock protein 70 expression in NIH3T3 cells [57]. These findings suggest that the 3% NaCl not only can activate PKC but also causes changes in gene expression mediated by the PKC in the cell. We used the PKC inhibitor of calphostin C to further identify the role of PKC in HS down-regulation of the expression of AQP4. Our results demonstrated that calphostin C attenuated the decrease of AQP4 expression induced by 3% NaCl in the primary astrocytes. The results indicate that 3% NaCl can attenuate the expression of AQP4 through activation of PKC.

## Conclusions

This study shows that osmotherapy with 3% NaCl ameliorated LPS-induced cerebral edema *in vivo*. In addition to its osmotic force, 3% NaCl exerted anti-edema effects possibly through down-regulating the expression of proinflammatory cytokines (IL-1β and TNFα) and inhibiting the expression of AQP4 induced by proinflammatory cytokines. Three percent NaCl attenuated the expression of AQP4 through activation of PKC in astrocytes. However, the optimal HS dose for reducing AQP4 expression, the pathway of activating PKC by HS and the significance of HS down-regulation of AQP4 in brain edema are to be further explored.

## Key messages

• 3% NaCl attenuated brain edema induced by LPS.

• 3% NaCl inhibited the increase of IL-1β and TNFα in brain tissues induced by LPS.

• 3% NaCl blocked the increase of AQP4 mRNA and protein in brain tissues induced by LPS *in vivo*.

• 3% NaCl blocked the increase of AQP4 mRNA and protein in the primary astrocytes induced by IL-1β through activation of PKC *in vitro*.

## Abbreviations

AQP4: aquaporin 4; BBB: blood-brain barrier; bp: base pair; BWC: brain water content; (D)MEM: (Dulbecco's) modified Eagle's medium; ELISA: enzyme-linked immunosorbent assay; FCS: fetal calf serum; HRP: horseradish peroxidase; HS: hypertonic saline; IgG: immunoglobulin G; IL-1β: interleukin-1 beta; LPS: lipopolysaccharide; NaCl: sodium chloride; PBS: phosphate-buffered saline; PKC: protein kinase C; RT-PCR: reverse transcriptase-polymerase chain reaction; TNFα: tumor necrosis factor alpha.

## Competing interests

The authors declare that they have no competing interests.

## Authors' contributions

CC participated in ELISA, flow cytometry, western blotting and real-time RT-PCR detection and drafted the manuscript. ZCL, NZ, HH and MW participated in the isolation and treatment of astrocytes and detection of brain water content. GJ and HS participated in the design of the study and performed the statistical analysis. SY, ZL and XY conceived of the study, and participated in its design and coordination and helped to draft the manuscript. All authors read and approved the final manuscript.
